# Human opinion dynamics: An inspiration to solve complex optimization problems

**DOI:** 10.1038/srep03008

**Published:** 2013-10-21

**Authors:** Rishemjit Kaur, Ritesh Kumar, Amol P. Bhondekar, Pawan Kapur

**Affiliations:** 1 CSIR-Central Scientific Instruments Organisation, Chandigarh, India; 2 Academy of Scientific & Innovative Research, New Delhi, India

## Abstract

Human interactions give rise to the formation of different kinds of opinions in a society. The study of formations and dynamics of opinions has been one of the most important areas in social physics. The opinion dynamics and associated social structure leads to decision making or so called opinion consensus. Opinion formation is a process of collective intelligence evolving from the integrative tendencies of social influence with the disintegrative effects of individualisation, and therefore could be exploited for developing search strategies. Here, we demonstrate that human opinion dynamics can be utilised to solve complex mathematical optimization problems. The results have been compared with a standard algorithm inspired from bird flocking behaviour and the comparison proves the efficacy of the proposed approach in general. Our investigation may open new avenues towards understanding the collective decision making.

Agglomeration in physical or behavioural space has been the essence of evolution. From simple biological systems such as single celled organisms to complex ones, such as humans, exhibit collective patterns emerging from simple interactions of large numbers of individuals. Several mathematical models have been proposed to explain the physical or behavioural agglomeration phenomenon in bacterial colonies, arthropods, pisces, avian, quadrupeds and humans. These models have been modified and exploited to be used in solving many complex problems ranging from dynamic routing, path finding to search strategies and mathematical optimization. Some examples include Ant Colony Optimization (ACO)^1^,^2^, Particle Swarm Optimization (PSO)^3^, Bat Algorithm (BA)^4^,^5^, Firefly Algorithm (FA)^6^, Bacteria Foraging Optimization (BFO)^7^ etc.

Surprisingly, in spite of several models describing the human interaction networks e.g. opinion dynamics, cultural dynamics, language dynamics, crowd behaviour, formation of hierarchies, human dynamics and social spreading phenomenon being proposed^8^, they have been seldom utilised for mathematical optimization and search strategies. Shi^9^ argues: "Human beings are social animals and are the most intelligent animals in the world. Therefore, it is natural to expect that an optimization algorithm inspired by human creative problem solving process will be a good optimization algorithm", suggesting use of human opinion formation models in the optimization domain.

An opinion can be defined as a degree of preference of an individual towards a particular phenomenon or thing. The opinion formation in a social network is an evolutionary process which is affected by the real or imagined presence of others^10^,^11^. Several theories have been put forward to understand the opinion dynamics or propagation of belief among individuals in these networks^12^ and there is no consensus among researchers on it, firstly, as opinions cannot be always modelled simply by binary states such as being ‘pro’ or ‘contra’, but they may take continuous values in an interval, secondly, their formation doesn't resemble discreet cascading but better resembles a process where consensus is reached by continuous influence from each other^13^. Modern societies exhibit a large degree of pluralism in social, political and cultural opinions. Moreover, it is evident that humans tend to agglomerate (form clusters) based upon opinion consensus and differences besides individualism (uniqueness in opinions), making the explanation of the coexistence of ‘global diversity’ and ‘opinion clustering’ complex and difficult. In order to mathematically model opinion, it has to be a variable or set of variables (collection of numbers) and if opinions can be represented by numbers, the real challenge is to find an adequate set of hypothesis to explain the mechanisms responsible for its evolution and formation^8^.

Human opinion formation models, particularly, Nowak–Szamrej–Latan'e models have been demonstrated for solving optimization problems^14^,^15^,^16^,^17^ and has been referred to as Social Impact Theory based Optimizer (SITO). However, these optimizers are based on discreet opinion formation and are limited to low dimensional real valued problems. Various continuous opinion models have been proposed and the most popular ones being Deffuant^8^,^18^ and Hegselmann-Krause^8^,^19^,^20^. These models typically assume ‘bounded confidence’ (BC) i.e. the interaction between the individuals is determined by a preset threshold value. Being deterministic in nature, the final state of a system after an interaction is always well defined, and results in early convergence of opinions in these models. To avoid this convergence, researchers experimented by adding an ‘opinion noise’ which in fact, is a practice in the binary opinion formation models. However, the addition of uniformly distributed opinion noise leads to sudden, large and unmotivated opinion change of individuals^21^.

The pioneering work of Durkheim^22^ towards understanding the division of labour in society originated from the quest to understand the relations of the individual to social solidarity. He argued on the parallelism between individualism and agglomeration in the society. He argued that integrating forces motivate individuals to conform and adopt values/norms similar to others, at the same time, disintegrative forces that encourage individualism threaten the social integration. Recently, Mas *et al.*^12^ simulated a model of social influence based on Durkheim's theory of social integration, that besides anomie and monoculture shows a pluralistic phase characterised by opinion clustering, i.e. it combines the integrative tendencies of social influence with the disintegrative effects of individualisation, which is achieved by incorporating an *adaptive noise*. These characteristics of Mas's computational model encouraged us to develop a real valued optimizer referred henceforth as the Continuous Opinion Dynamics Optimizer (CODO). The algorithm is governed by four basic rudiments namely *social structure*, *opinion space*, *social influence* and *updating rule* as explained below.

*Social structure* is an important aspect of social dynamics which governs the interaction between two individuals, among individuals, the frequency of interactions and the way of interactions. Many different social structures e.g. small world^23^,^24^, random graphs^25^,^26^, cellular automata model^27^ etc. have been proposed and simulated in social physics. The individuals form the nodes of a social graph and the edges define the neighbourhood set of an individual with whom it interacts. In this work, we have considered a simplistic model of cellular automata in which an individual is characterized by a cell and its neighbourhood is determined according to Moore topology.

The second basic rudiment of the proposed algorithm is *Opinion space.* In social physics terms, as already described, the individual's opinion may be of two kinds: discrete or continuous. Discrete opinions may take values such as {0,1} or {−1,1}, whereas, continuous opinions could be any real value. Each individual *i* is characterized by an opinion vector ***o_i_(t)***, at a particular time *t*. This setting enables us to search in a multidimensional space. The opinion vectors of all individuals are initialized randomly using a uniform distribution.

Decision making process of an individual in a society is governed by his own considerations or/and the opinions of others. Opinions, in general, are formed by the direct or indirect influence of cultural norms, interactions and mass media. *Social influence* is the combined effect of these influences, due to which, individuals act in accordance to the beliefs and expectations of others. This forms the third rudiment of the algorithm. However, for the simplicity of modelling, only local dynamics representing the social influence has been considered in this work. Therefore, the social influence has been formulated by considering two factors i.e. distance between two individuals and the social ranking (*SR*) of the individuals^11^. The *SR* of the individuals is determined by their respective fitness values. These fitness values are the output values of the objective function to be minimized. The individual with the minimum fitness value is assigned the highest *SR*, the highest possible *SR* being the total number of individuals. The individuals with same fitness values are assigned the same *SR*.

The social influence *w_ij_(t)* of individual *j* on individual *i* is given by equation (1)

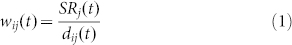
where *d_ij_(t)* is the Euclidean distance between individuals *i* and *j*.

One of the important elements of any iterative optimization algorithm is it's *Updating rule* which governs its dynamics in general. The social interaction models invariably encompass the idea of change of opinions of an individual. Various strategies/rules have been adopted to update the opinions, as stated in the literature^11^,^18^,^19^,^20^,^28^. However, the updating rule in the context of optimization problems determines the new position of individuals in the search space. As already discussed above, update rule based on Durkheimian opinion dynamics has been used here. The update rule can be put according to equation (2)

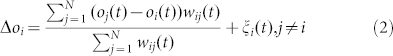
Where, ***o_j_(t)*** is the opinion of neighbours of individual *i*, *N* is the no. of neighbours, *w_ij_(t)* represents the social influence and *ξ_i_(t)* is a normally distributed random noise with mean zero and standard deviation *σ_i_(t)*

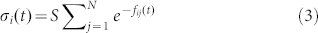
where, *S* denotes the strength of disintegrating forces in the society and *f_ij_(t)* denotes the modulus of difference in fitness values of individual *i* and individual *j* at time *t*. The higher the value of *σ_i_(t)*, the higher is the tendency of an individual towards individualization. This setting follows the Durkheim's theory, so as the individual's tendency towards individualisation increases as the number of individuals with similar fitness increases. This adaptive mechanism prevents the algorithm from reaching early convergence and hence, *ξ_i_(t)* is also referred to as *adaptive noise*^12^.

Figure 1 shows the simplified schema of the CODO process. At particular time/iteration *t*, the opinion vectors of all the individuals are evaluated by the objective function. The fitness values (output value of the objective function) thus obtained are used to assign a social rank to each individual. The lower the fitness value, the higher the rank and vice versa. Individuals with the same fitness values attain the same social rank. The social influence of each individual is then determined using equation (1). At the end of each iteration, the opinion vector of each individual is updated. This iterative process terminates after attaining preset fitness error value or maximum number of objective function evaluations. The pseudo code for CODO is given in the Methods section.

The proposed optimizer has been tested on 28 benchmark functions for 10 and 30 dimensions and compared with local best PSO (lbest PSO)^29^, one of the variants of popular PSO. lbest PSO has been chosen for comparison due to its close structural similarity to the proposed algorithm, in general, the proposed algorithm performs better as compared to lbest PSO. The algorithm has been investigated for the effects of disintegrative forces in the society. The results suggest that, the interplay and balance of integrative and disintegrative forces in a society may be utilised for solving complex mathematical problems.

## Results

This section presents the experiments that have been used to test the efficacy of the proposed algorithm. First, we describe the various benchmark functions used to test the algorithm then we describe the parameters and settings that have been used in the configuration of our algorithm. Further, we compare the CODO algorithm with lbest PSO algorithm. For the fair comparison, lbest PSO was implemented in the same topology as CODO. i.e. Von-Neumann with Moore neighbourhood. At last, we explore the effect of disintegrative forces on solving the optimization problem.

In order to analyze the proposed algorithm we have selected the set of problems proposed for CEC competition^30^ on real-parameter optimization in 2013. The benchmark suite consists of 28 objective functions (see Supplementary Table S1). The detailed mathematical description of the functions is present in^30^. All the test functions are minimization problems defined as: min *f(x)*, ***x*** = *[x_1_, x_2_, x_3_, … x_D_]^T^*, where D = Dimension of the problem and ***ô*** = *[ô_1_, ô_2_, ô_3_, … ô_D_]^T^* is the global optimum which is shifted and randomly distributed in [−80, 80]^D^. As suggested in the document, the search range is [−100, 100]^D^. There are three broad classes of functions provided in the test suite, unimodal, basic multimodal and composite functions. F1 to F5 are unimodal and rest are multimodal functions, F21 to F28 are composition functions. These functions have several different properties such as multi-modality, non-separability, deceptiveness, asymmetricity etc and hence are difficult to solve. All the functions have been rotated and shifted in order to not let the users do a biased search. We have considered D = 10 and 30 for all the problems. For the entire test purpose we considered the functions as black box problems i.e. we did not tune the parameters of the proposed algorithm individually for each function. The termination condition for the algorithm has been set to Maximum Function Evaluation (MaxFES) i.e. 10000*D, or minimum error of 10^−8^, whichever occurs first. As suggested, 51 runs per problem were performed. These runs were performed on machines of different configurations.

The parameters used in our proposed algorithm are summarized in Table 1. The behaviour of the algorithm using a society size of 25 and 36 for 10 and 30 dimensions respectively, has been studied. The individuals have been arranged in a 2D grid like structure and Moore neighbourhood of 2 has been used. The *S* factor has been set to 8 (determined empirically) for all the experiments. The initial opinions of society has been scattered randomly in search space of [−100, 100]^D^.

### Unimodal functions

**Figure 2:**
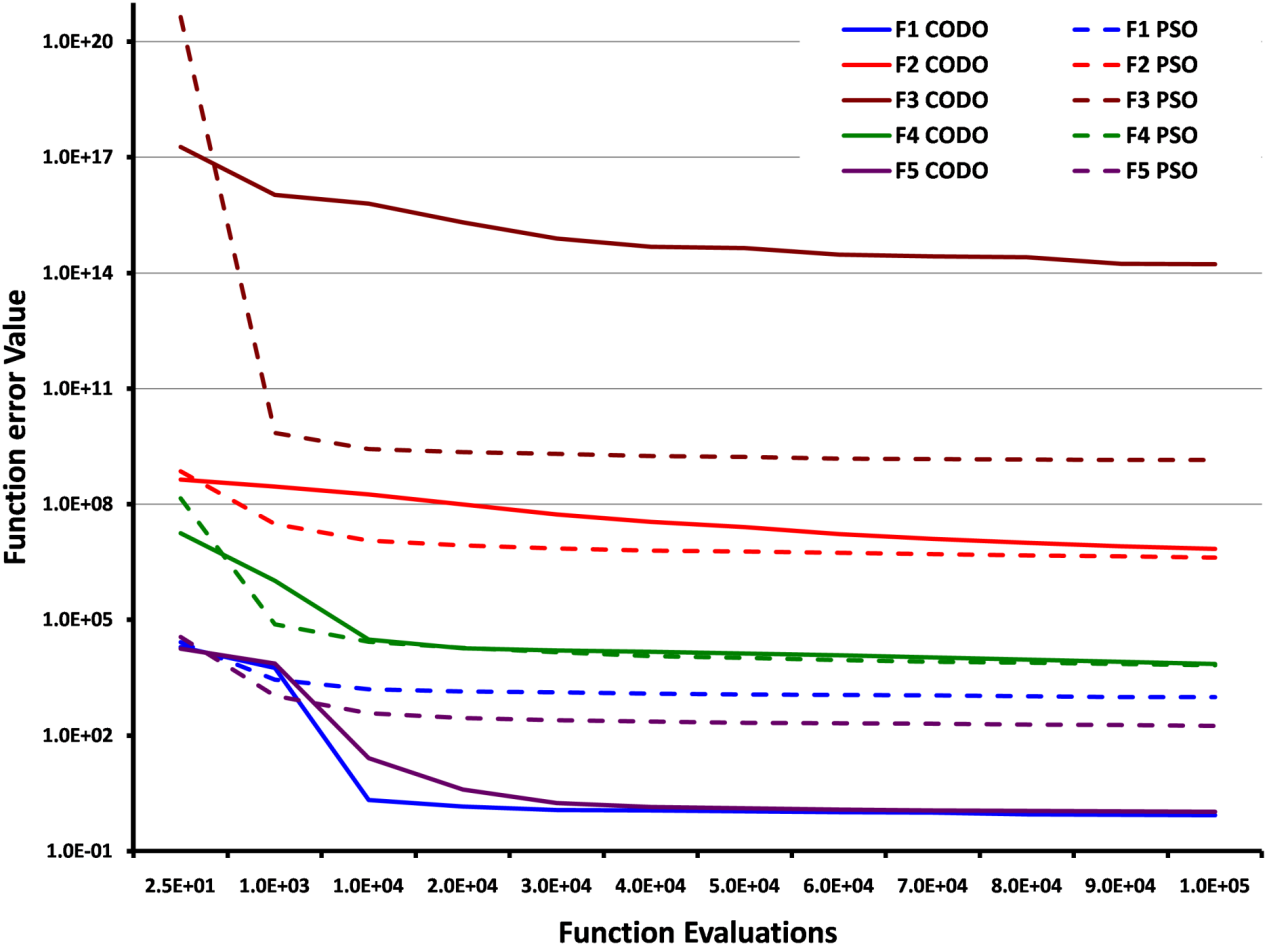
Convergence rate for unimodal functions. This figure shows the function error value vs. function evaluations of CODO and lbest PSO for all the unimodal (F1–F5) functions. The convergence rate of each function is represented in different colors. The dotted lines show lbest PSO results, whereas CODO results are represented by solid lines. The decreasing trend of lines shows the convergence towards the minimum.

Figure 2 shows the function error value vs. function evaluations of CODO and lbest PSO for all the unimodal (F1–F5) functions. Wherein, relatively poor performance for F2 and F3 is observable due to the inherent characteristics of these functions (i.e. F2 is ill-conditioned function and F3 has a peculiar smooth but narrow ridge), although a steady decrease in fitness value suggests further convergence. Also, it is observed that CODO performs better than lbest PSO in case of F1 and F5, whereas similar performance is obtained in case of F4. lbest PSO performs better than CODO in case of F2 and F3. A minimum error value in order of 10^−1^ has been achieved at the end of MaxFES for F1 and F5.

### Basic multimodal functions

**Figure 3:**
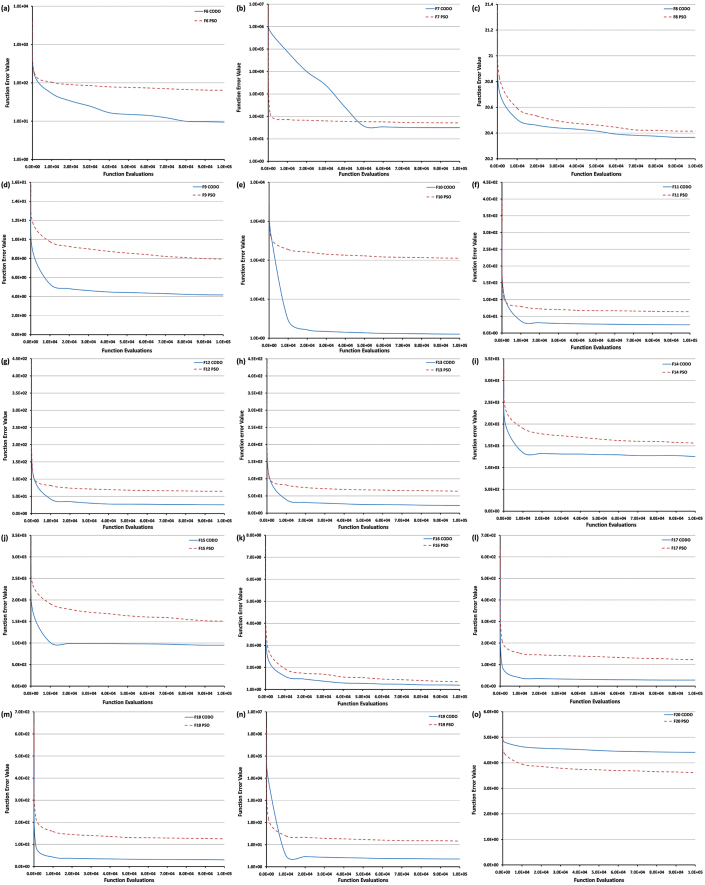
Convergence rate for basic multimodal functions. This figure shows the function error value vs. function evaluations of the algorithm for all the basic multimodal functions (F6–F20). The convergence rate of each function is represented in different colors. The dotted lines show lbest PSO results, whereas CODO results are represented by solid lines. The decreasing trend of lines shows the convergence towards the minimum.

Figure 3(a–o) shows the function error value vs. function evaluations of the algorithm for all the basic multimodal functions (F6–F20). One of the important measures to assess the performance of an algorithm is its invariance to translation and angle preserving (rigid) transformations of the search space (translation, rotation). It is observed that F11 (Rastrigin's function) & F12 (Rotated Rastrigin's function), F14 (Schwefel's function) & F15 (Rotated Schwefel's function) and F17 (Lunacek Bi_Rastrigin function) & F18 (Rotated Lunacek Bi_Rastrigin function) show similar convergence results, hence proving the rotation invariance property of the CODO. Also, the convergence results for F12 and F13 are similar in spite of F13 being a non-continuous version of F12. This proves the efficacy of algorithm in case of non continuous cases as well. It is observed that CODO performs better in all the functions except F20.

### Composition functions

**Figure 4:**
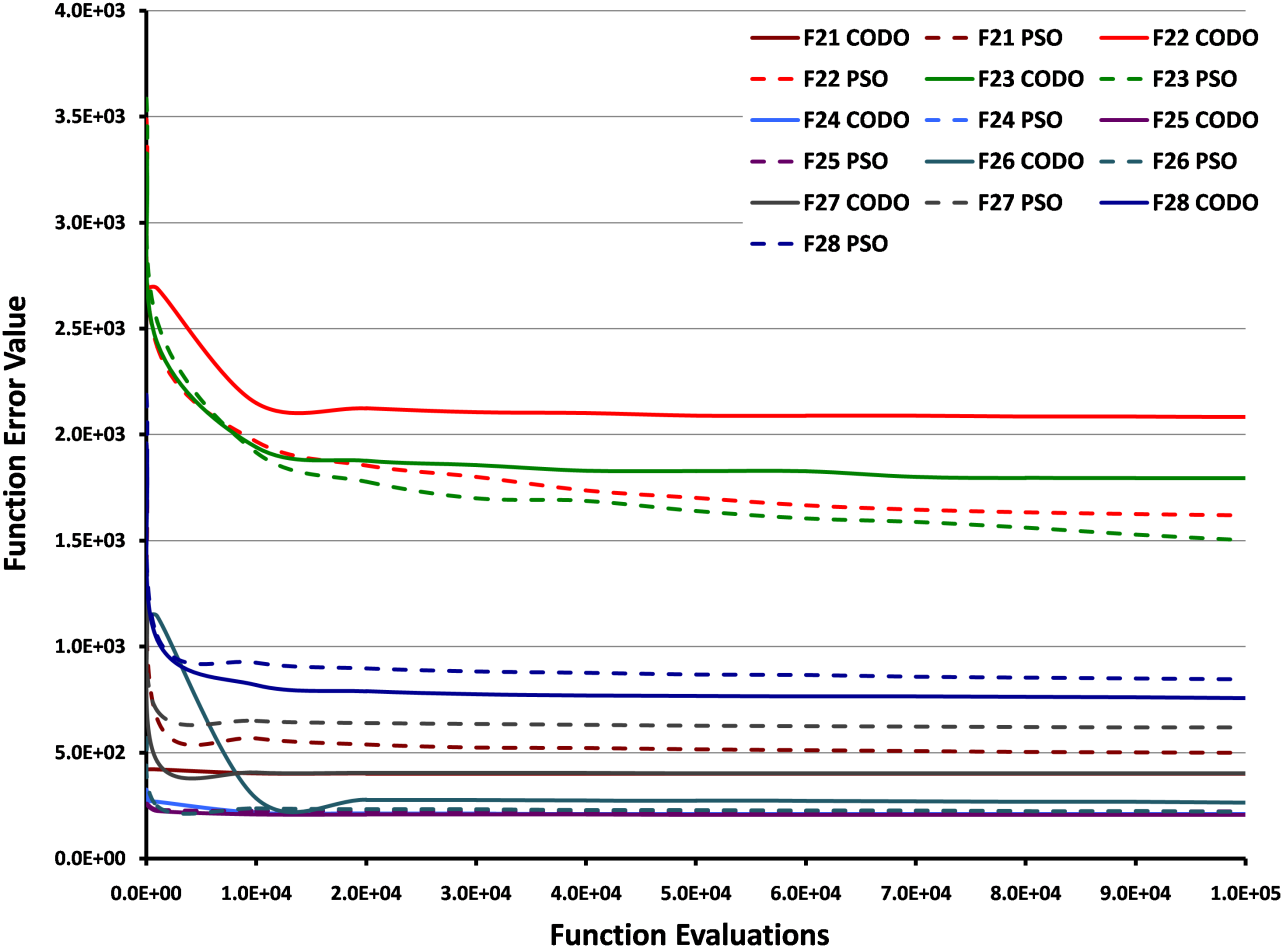
Convergence rate for composition functions. This figure shows the function error value vs function evaluations of the algorithm for all the composition functions (F21–F28). The convergence rate of each function is represented in different colors. The dotted lines show lbest PSO results, whereas CODO results are represented by solid lines. The decreasing trend of lines shows the convergence towards the minimum.

Figure 4 shows the function error value vs function evaluations of the algorithm for all the composition functions (F21–F28). CODO and lbest PSO did not perform well on functions F22 and F23. This could be attributed to the fact that the basic function used in construction of these functions is Schwefel's function (F14) and rotated Schwefel's function (F15) respectively, on which both the algorithms did not perform very well in general. For further statistical details of comparison results, see Supplementary Tables S2 and S3.

### Effect of disintegrative forces

**Figure 5:**
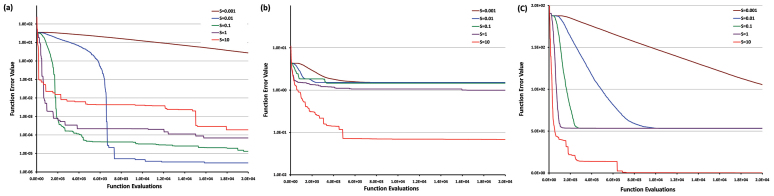
Effect of disintegrative forces. This figure shows the convergence trends of (a) unimodal function (F1) (b) basic multimodal function (F11) (c) composition function (F21) for [0.001,0.01, 0.1,1, 10] values of *S*. Convergence results for different values of *S* are shown in different colours.

Disintegrative forces play a key role in the evolution of opinion formation in a society. Stronger disintegrative forces encourage individualisation tendencies leading to “anomie” (a state of extreme individualism without a social structure). Similarly, weak disintegrative forces result in monoculture. Therefore, balancing the integrative and disintegrative forces in order to maintain pluralism without endangering clustering becomes important from optimization point of view. The parameter *S* enables us to vary the strength of the disintegrating forces in the society. Increasing *S*, increases the standard deviation of the distribution from which the adaptive noise is drawn, thereby increasing the disintegrating forces. In terms of evolutionary optimization jargon, the strength of disintegrating forces determines the “exploration” and “exploitation” capabilities of the algorithm. Wherein, “exploration” refers to the global search and “exploitation” is fine tuning in the local vicinity. This compels us to empirically determine the optimum value of *S*. Although, fine tuning of this parameter is problem specific, the values of *S* were set to [0.001, 0.01, 0.1,1, 10] in general, to study the effects of the disintegrating forces. Figure 5 (a, b and c) shows the convergence trends of functions from unimodal (F1), basic multimodal (F11) and composition (F21) categories for the aforesaid values of *S* respectively. Function F1 has been chosen for its simplicity, F11 for its popularity and F21 randomly as all the functions in this category (i.e. composition) have similar properties. It may be noted that these results have been obtained on two dimensions for 20,000 MaxFES and 51 runs. In case of Fig. 5(a) it may be observed that although for all values of *S* convergence to the optima is guaranteed, the rate of convergence is governed by its value. In general, for unimodal functions we get higher precision for the lower value of *S*. Although the optimum value of *S* in this case can be said to be 0.01 but setting a value of *S* is problem specific. For multimodal functions (Fig. 5(b and c)), the larger the value of *S*, the better chances we have of reaching global minima. This may be attributed to the fact that larger *S* value brings with itself more diversity (greater individualisation) in the society and hence, the probability of getting stuck in local minima is less. Whereas, for lower values of *S*, the individuals tend to quickly gravitate towards consensus and get stuck in local minima. However, schemes facilitating the dynamic changes in *S* may be incorporated in order to achieve faster convergence and precision.

## Discussion

In this paper, we showed that human opinion formations and their interaction dynamics can be used to solve complex mathematical problems. Although, from a social physics point of view these opinion dynamics models are very naive and limited in nature but we feel the present research will pave a path towards developing novel methods and tools to understand the real world problem solving by human beings in a social structure. Later, the effect of social structure can also be studied viz. small world, scale free and random graphs etc. The effect of evolution of network with time could also be one of the areas to be studied. We also showed the effect of adaptive noise in the algorithm and compared the overall performance with lbest PSO. It is worthwhile to note that the proposed algorithm has a single control parameter (*S*) unlike other optimizers, making it easier to tune. Although, the algorithm in its present form is very basic and does not perform well in the case of highly multimodal problems, but it can be studied and adapted to improve its performance by evolving strategies to dynamically update *S* parameter to attain finer grained search.

## Methods

The pseudo-code of the CODO algorithm is as follows:

1. Initialize opinions for every dimension d by random assignment of values from (Xmin, Xmax) to society.opinions;
2. *iter* = 0;
3. WHILE (iter < max_iter && error > = min_error) DO
4. society.fitness = EvaluateFitnessFcn(society.opinions);
5. society.ranking = calcRank(society.fitness); // It ranks the individuals based on society fitnessiter = iter + 1;
6. FOR each individual i and each dimension d DO
7. Calculate *w_ij_* of neighbours j of individual i with respect to dimension d.
8. Update opinion of individual i as defined above.

END (FOR)

END (WHILE)

## Author Contributions

^1^R.K. devised the model and performed the research. ^1^R.K., ^2^R.K. and A.P.B. analyzed the results. A.P.B. prepared the figures. ^2^R.K. and A.P.B. wrote the main text of manuscript. ^1^R.K., ^2^R.K., A.P.B. and P.K. reviewed the manuscript.

## Supplementary Material

Supplementary Information Supplementary Document

## Figures and Tables

**Figure 1:**
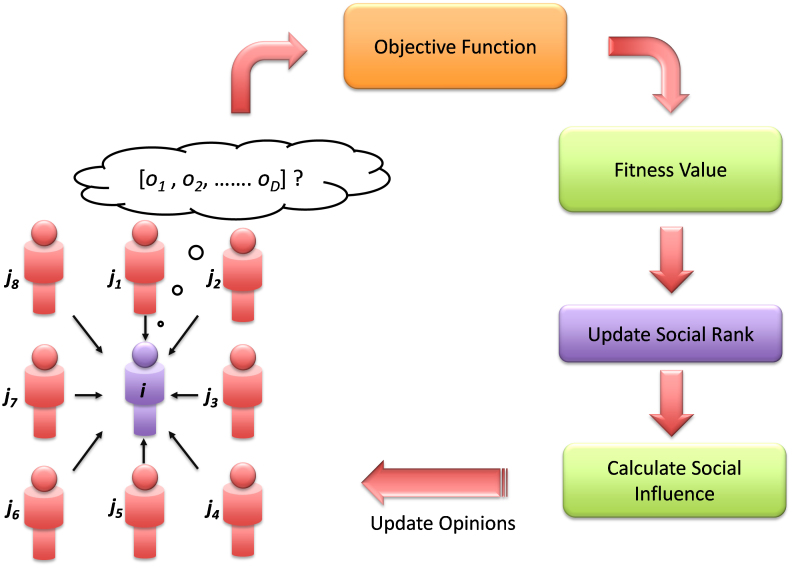
CODO schema. This figure shows the optimization process of CODO.

**Table 1:** Parameters settings. This table shows values of parameters for CODO and lbest PSO

Society Structure	2D cellular grid
Society Size	25, D = 10 36, D = 30
Neighbourhood	Moore configuration of size 2
Disintegrative parameter(*s*)	8
Initialization	Uniform random distribution
MaxFES	10000*D
Stopping criterion	Find optimum with minimum error of 10^−8^ or MaxFES
No of runs	51
Search range	[−100, 100]
Inertia weight	1
Acceleration coefficients	[2,2]
