# Supplementary material for: Human opinion dynamics: An inspiration to solve complex optimization problems
Source: Sci Rep. 2013 Oct 21;3:3008. doi: 10.1038/srep03008 (PMC3801113; doi:10.1038/srep03008)
Supplement: Supplementary Information — Supplementary Document [file srep03008-s1.doc]

Supporting Online Material

*Human opinion dynamics: an inspiration to solve complex optimization problems*

by Rishemjit Kaur, Ritesh Kumar, Amol P Bhondekar, Pawan Kapur

| **Supplementary Table S1: Function List**  This table lists the set of problems proposed for CEC competition^1^ on real-parameter optimization in 2013. The benchmark suite consists of 28 objective functions. The detailed mathematical description of the functions is present in ^1^ | | | |
| --- | --- | --- | --- |
|  | **No.** | **Functions** | ***f_i_* = f_i_*(*x**)** |
| **Unimodal Functions** | F1 | Sphere Function | -1400 |
|  | F2 | Rotated High Conditioned Elliptic Function | -1300 |
|  | F3 | Rotated Bent Cigar Function | -1200 |
|  | F4 | Rotated Discus Function | -1100 |
|  | F5 | Different Powers Function | -1000 |
| **Basic Multimodal Functions** | F6 | Rotated Rosenbrock’s Function | -900 |
|  | F7 | Rotated Schaffers F7 Function | -800 |
|  | F8 | Rotated Ackley’s Function | -700 |
|  | F9 | Rotated Weierstrass Function | -600 |
|  | F10 | Rotated Griewank’s Function | -500 |
|  | F11 | Rastrigin’s Function | -400 |
|  | F12 | Rotated Rastrigin’s Function | -300 |
|  | F13 | Non-Continuous Rotated Rastrigin’s Function | -200 |
|  | F14 | Schwefel's Function | -100 |
|  | F15 | Rotated Schwefel's Function | 100 |
|  | F16 | Rotated Katsuura Function | 200 |
|  | F17 | Lunacek Bi_Rastrigin Function | 300 |
|  | F18 | Rotated Lunacek Bi_Rastrigin Function | 400 |
|  | F19 | Expanded Griewank’s plus Rosenbrock’s Function | 500 |
|  | F20 | Expanded Scaffer’s F6 Function | 600 |
| **Composition Functions** | F21 | Composition Function 1 (n=5,Rotated) | 700 |
|  | F22 | Composition Function 2 (n=3,Unrotated) | 800 |
|  | F23 | Composition Function 3 (n=3,Rotated) | 900 |
|  | F24 | Composition Function 4 (n=3,Rotated) | 1000 |
|  | F25 | Composition Function 5 (n=3,Rotated) | 1100 |
|  | F26 | Composition Function 6 (n=5,Rotated) | 1200 |
|  | F27 | Composition Function 7 (n=5,Rotated) | 1300 |
|  | F28 | Composition Function 8 (n=5,Rotated) | 1400 |

The function error value (*f_i_*(*x*)-*f_i_*(*x**)) was recorded after (0.01, 0.1, 0.2, 0.3, 0.4, 0.5, 0.6, 0.7, 0.8, 0.9, 1.0)*MaxFES for each run. Table S2 and S3, show the best, worst, mean, median and standard deviationvalues of the function errors for 51 runs of CODO and lbest PSO. Table 2 and 3 represent the results for 10D and 30D, respectively.

| **Supplementary Table S2: Comparison of error values obtained for 10 Dimensions**  This table shows the best, worst, mean, median and standard deviationvalues of the function errors for 51 runs of CODO and lbest PSO for 10D. The function error value (*f_i_*(*x*)-*f_i_*(*x**)) was recorded after (0.01, 0.1, 0.2, 0.3, 0.4, 0.5, 0.6, 0.7, 0.8, 0.9, 1.0)*MaxFES for each run. | | | | | | | | | | | |
| --- | --- | --- | --- | --- | --- | --- | --- | --- | --- | --- | --- |
|  | **CODO** | | | | |  | **lbest PSO** | | | | |
| **Function** | **Best** | **Worst** | **Median** | **Mean** | **Std. Dev** |  | **Best** | **Worst** | **Median** | **Mean** | **Std. Dev** |
| 1 | 4.2626E-01 | 1.3959E+00 | 8.1600E-01 | 8.5037E-01 | 2.3244E-01 |  | 4.9471E+02 | 1.4317E+03 | 1.0321E+03 | 9.9280E+02 | 2.2536E+02 |
| 2 | 1.1928E+06 | 3.3411E+07 | 5.1179E+06 | 6.9852E+06 | 6.4057E+06 |  | 6.3653E+05 | 1.2309E+07 | 4.2245E+06 | 4.1335E+06 | 2.0782E+06 |
| 3 | 2.4835E+09 | 5.8984E+15 | 2.0890E+12 | 1.6990E+14 | 8.4446E+14 |  | 6.6434E+08 | 1.9948E+09 | 1.4156E+09 | 1.3945E+09 | 3.5144E+08 |
| 4 | 2.8913E+03 | 1.4351E+04 | 6.6827E+03 | 7.2271E+03 | 2.7801E+03 |  | 2.8912E+03 | 1.3267E+04 | 6.5601E+03 | 6.6083E+03 | 2.2714E+03 |
| 5 | 6.7640E-01 | 1.5813E+00 | 1.0478E+00 | 1.0581E+00 | 2.0550E-01 |  | 1.0871E+02 | 2.8049E+02 | 1.6414E+02 | 1.7597E+02 | 4.4874E+01 |
| 6 | 4.7093E-01 | 1.2021E+01 | 1.1004E+01 | 9.4784E+00 | 3.7615E+00 |  | 3.7705E+01 | 1.1124E+02 | 6.2021E+01 | 6.4090E+01 | 1.6267E+01 |
| 7 | 3.7943E+00 | 8.4988E+01 | 2.6856E+01 | 3.1389E+01 | 1.8244E+01 |  | 2.8073E+01 | 6.5840E+01 | 5.2828E+01 | 5.1495E+01 | 8.9296E+00 |
| 8 | 2.0211E+01 | 2.0501E+01 | 2.0382E+01 | 2.0366E+01 | 7.2687E-02 |  | 2.0176E+01 | 2.0620E+01 | 2.0425E+01 | 2.0415E+01 | 9.7903E-02 |
| 9 | 2.5408E+00 | 6.1845E+00 | 4.2601E+00 | 4.1699E+00 | 7.7938E-01 |  | 5.8400E+00 | 9.3146E+00 | 8.0078E+00 | 7.9344E+00 | 7.5834E-01 |
| 10 | 1.0124E+00 | 1.5220E+00 | 1.2667E+00 | 1.2711E+00 | 1.0795E-01 |  | 5.5495E+01 | 1.5543E+02 | 1.1151E+02 | 1.1279E+02 | 2.5854E+01 |
| 11 | 8.9933E+00 | 4.7977E+01 | 2.4199E+01 | 2.4688E+01 | 8.8246E+00 |  | 3.8691E+01 | 7.5714E+01 | 6.4518E+01 | 6.3712E+01 | 7.7426E+00 |
| 12 | 1.1481E+01 | 4.8337E+01 | 2.4960E+01 | 2.5256E+01 | 7.5461E+00 |  | 4.8164E+01 | 7.9289E+01 | 6.5124E+01 | 6.4764E+01 | 7.2744E+00 |
| 13 | 7.6074E+00 | 4.0054E+01 | 2.2309E+01 | 2.2216E+01 | 8.1788E+00 |  | 4.2684E+01 | 7.8193E+01 | 6.5955E+01 | 6.4049E+01 | 7.3501E+00 |
| 14 | 3.7476E+02 | 1.8502E+03 | 1.3155E+03 | 1.2546E+03 | 3.1509E+02 |  | 1.1929E+03 | 1.8991E+03 | 1.5744E+03 | 1.5633E+03 | 1.8156E+02 |
| 15 | 3.0413E+02 | 1.5346E+03 | 8.7137E+02 | 9.5022E+02 | 2.9086E+02 |  | 8.0857E+02 | 1.8314E+03 | 1.5588E+03 | 1.5091E+03 | 1.9312E+02 |
| 16 | 6.7904E-01 | 1.5274E+00 | 1.2299E+00 | 1.1940E+00 | 2.0926E-01 |  | 4.2058E-01 | 1.9812E+00 | 1.4050E+00 | 1.3496E+00 | 2.8487E-01 |
| 17 | 1.9140E+01 | 3.9117E+01 | 2.8600E+01 | 2.8069E+01 | 4.5856E+00 |  | 8.6829E+01 | 1.5729E+02 | 1.2623E+02 | 1.2300E+02 | 1.5571E+01 |
| 18 | 2.1772E+01 | 3.7975E+01 | 3.0313E+01 | 2.9982E+01 | 3.9034E+00 |  | 7.4365E+01 | 1.6792E+02 | 1.2892E+02 | 1.2484E+02 | 1.8009E+01 |
| 19 | 1.1387E+00 | 3.4089E+00 | 2.2769E+00 | 2.2391E+00 | 3.9687E-01 |  | 9.5157E+00 | 2.4672E+01 | 1.3882E+01 | 1.4577E+01 | 3.4610E+00 |
| 20 | 3.9512E+00 | 4.4521E+00 | 4.1289E+00 | 4.1417E+00 | 1.4130E-01 |  | 2.9346E+00 | 3.8525E+00 | 3.6547E+00 | 3.6093E+00 | 1.8345E-01 |
| 21 | 4.0035E+02 | 4.0062E+02 | 4.0044E+02 | 4.0046E+02 | 6.6431E-02 |  | 4.3986E+02 | 5.5055E+02 | 5.0484E+02 | 4.9920E+02 | 2.5676E+01 |
| 22 | 1.3979E+03 | 2.6397E+03 | 2.1206E+03 | 2.0834E+03 | 2.8213E+02 |  | 1.2178E+03 | 2.0732E+03 | 1.6608E+03 | 1.6193E+03 | 1.8837E+02 |
| 23 | 8.5177E+02 | 2.4403E+03 | 1.8262E+03 | 1.7949E+03 | 3.6694E+02 |  | 1.0510E+03 | 1.8786E+03 | 1.5148E+03 | 1.5004E+03 | 1.7862E+02 |
| 24 | 1.2952E+02 | 2.2604E+02 | 2.1579E+02 | 2.0948E+02 | 2.0272E+01 |  | 2.1672E+02 | 2.2718E+02 | 2.2173E+02 | 2.2176E+02 | 2.2984E+00 |
| 25 | 2.0336E+02 | 2.2151E+02 | 2.0423E+02 | 2.0630E+02 | 5.0726E+00 |  | 2.1739E+02 | 2.3001E+02 | 2.2123E+02 | 2.2163E+02 | 2.5260E+00 |
| 26 | 1.1308E+02 | 4.1101E+02 | 3.1246E+02 | 2.6454E+02 | 1.0608E+02 |  | 1.3872E+02 | 3.2597E+02 | 2.0027E+02 | 2.2123E+02 | 6.6145E+01 |
| 27 | 3.1826E+02 | 4.0565E+02 | 4.0392E+02 | 4.0226E+02 | 1.2014E+01 |  | 5.5203E+02 | 6.5090E+02 | 6.2548E+02 | 6.1817E+02 | 2.3837E+01 |
| 28 | 3.1437E+02 | 1.0256E+03 | 7.8302E+02 | 7.5730E+02 | 1.4276E+02 |  | 3.6584E+02 | 9.1805E+02 | 8.5931E+02 | 8.4483E+02 | 7.8681E+01 |
|  | | | | | | | | | | | |
| **Supplementary Table S3: Comparison of error values obtained for 30 Dimensions**  This table shows the best, worst, mean, median and standard deviationvalues of the function errors for 51 runs of CODO and lbest PSO for 30D. The function error value (*f_i_*(*x*)-*f_i_*(*x**)) was recorded after (0.01, 0.1, 0.2, 0.3, 0.4, 0.5, 0.6, 0.7, 0.8, 0.9, 1.0)*MaxFES for each run. | | | | | | | | | | | |
|  | **CODO** | | | | |  | **lbest PSO** | | | | |
| **Function** | **Best** | **Worst** | **Median** | **Mean** | **Std. Dev** |  | **Best** | **Worst** | **Median** | **Mean** | **Std. Dev** |
| 1 | 5.5928E+00 | 1.1951E+01 | 8.0290E+00 | 8.0178E+00 | 1.3829E+00 |  | 7.3901E+03 | 1.2072E+04 | 1.0685E+04 | 1.0467E+04 | 1.0559E+03 |
| 2 | 1.3711E+08 | 6.9334E+08 | 2.8363E+08 | 3.0306E+08 | 1.2424E+08 |  | 6.1887E+07 | 2.0536E+08 | 1.1653E+08 | 1.2426E+08 | 3.2666E+07 |
| 3 | 4.0871E+14 | 1.7574E+19 | 4.0415E+16 | 6.8230E+17 | 2.5629E+18 |  | 1.2190E+10 | 3.0097E+10 | 2.1802E+10 | 2.1313E+10 | 3.6616E+09 |
| 4 | 5.6458E+03 | 2.7706E+04 | 1.1061E+04 | 1.1952E+04 | 4.2173E+03 |  | 1.7660E+04 | 3.5435E+04 | 2.5850E+04 | 2.6206E+04 | 4.3483E+03 |
| 5 | 4.3077E+00 | 9.7102E+00 | 7.2322E+00 | 7.1793E+00 | 1.2862E+00 |  | 1.7838E+03 | 3.6422E+03 | 3.0061E+03 | 2.9320E+03 | 4.4273E+02 |
| 6 | 1.1482E+01 | 9.6755E+01 | 7.8589E+01 | 7.1278E+01 | 1.8904E+01 |  | 5.0510E+02 | 8.2936E+02 | 6.6051E+02 | 6.6756E+02 | 6.7194E+01 |
| 7 | 2.9563E+01 | 5.1928E+05 | 7.7907E+01 | 1.0258E+04 | 7.2703E+04 |  | 9.4853E+01 | 1.5180E+02 | 1.2832E+02 | 1.2723E+02 | 1.2526E+01 |
| 8 | 2.0815E+01 | 2.1045E+01 | 2.0970E+01 | 2.0962E+01 | 4.0633E-02 |  | 2.0852E+01 | 2.1088E+01 | 2.1000E+01 | 2.0998E+01 | 5.0568E-02 |
| 9 | 1.6662E+01 | 2.8642E+01 | 2.0131E+01 | 2.0882E+01 | 3.0304E+00 |  | 3.3508E+01 | 4.1967E+01 | 3.8635E+01 | 3.8318E+01 | 1.9792E+00 |
| 10 | 3.7747E+00 | 7.6372E+00 | 5.1316E+00 | 5.2758E+00 | 8.9825E-01 |  | 8.3299E+02 | 1.6874E+03 | 1.2784E+03 | 1.2639E+03 | 1.9149E+02 |
| 11 | 1.7393E+02 | 3.1553E+02 | 2.3526E+02 | 2.3615E+02 | 3.4480E+01 |  | 2.9611E+02 | 3.6961E+02 | 3.4631E+02 | 3.4472E+02 | 1.6853E+01 |
| 12 | 1.3399E+02 | 3.0427E+02 | 1.9952E+02 | 1.9974E+02 | 3.6110E+01 |  | 2.6831E+02 | 3.8776E+02 | 3.5567E+02 | 3.5110E+02 | 2.1347E+01 |
| 13 | 7.2171E+01 | 2.3253E+02 | 1.5594E+02 | 1.5375E+02 | 3.1427E+01 |  | 3.2351E+02 | 3.8918E+02 | 3.5843E+02 | 3.5593E+02 | 1.4810E+01 |
| 14 | 3.2959E+03 | 5.9086E+03 | 4.7675E+03 | 4.7521E+03 | 6.1271E+02 |  | 6.8723E+03 | 8.0880E+03 | 7.4872E+03 | 7.4874E+03 | 2.7765E+02 |
| 15 | 3.1793E+03 | 5.7308E+03 | 4.5704E+03 | 4.6360E+03 | 5.8276E+02 |  | 6.3517E+03 | 8.1153E+03 | 7.5946E+03 | 7.4971E+03 | 3.6177E+02 |
| 16 | 1.8306E+00 | 3.0129E+00 | 2.4552E+00 | 2.4155E+00 | 3.1942E-01 |  | 1.6564E+00 | 3.4210E+00 | 2.9254E+00 | 2.8922E+00 | 3.3084E-01 |
| 17 | 1.3168E+02 | 2.2359E+02 | 1.6269E+02 | 1.6508E+02 | 1.7966E+01 |  | 5.8091E+02 | 8.3148E+02 | 7.0952E+02 | 7.0328E+02 | 5.3376E+01 |
| 18 | 1.5666E+02 | 2.1703E+02 | 1.7999E+02 | 1.7997E+02 | 1.1659E+01 |  | 5.2492E+02 | 7.8260E+02 | 6.8989E+02 | 6.9180E+02 | 5.2636E+01 |
| 19 | 1.0596E+01 | 1.7296E+01 | 1.4933E+01 | 1.4891E+01 | 1.3011E+00 |  | 3.2599E+02 | 2.7885E+03 | 1.3534E+03 | 1.3666E+03 | 5.1685E+02 |
| 20 | 14.5050E+00 | 14.9999E+0 | 14.9564E+0 | 14.9119E+0 | 0.1307E+00 |  | 1.2680E+01 | 1.5000E+01 | 1.5000E+01 | 1.4499E+01 | 8.2866E-01 |
| 21 | 1.5254E+02 | 4.5407E+02 | 3.5117E+02 | 3.7734E+02 | 5.8153E+01 |  | 1.4190E+03 | 2.2333E+03 | 2.0344E+03 | 1.9871E+03 | 1.9772E+02 |
| 22 | 5.3573E+03 | 8.2260E+03 | 7.0393E+03 | 6.9591E+03 | 6.8271E+02 |  | 6.7398E+03 | 8.0856E+03 | 7.5933E+03 | 7.6063E+03 | 3.0576E+02 |
| 23 | 5.3935E+03 | 7.4663E+03 | 6.6077E+03 | 6.5872E+03 | 4.7776E+02 |  | 6.4932E+03 | 8.4645E+03 | 7.5892E+03 | 7.5655E+03 | 3.9411E+02 |
| 24 | 2.1689E+02 | 3.2072E+02 | 2.2047E+02 | 2.2767E+02 | 2.3395E+01 |  | 2.9285E+02 | 3.0952E+02 | 3.0212E+02 | 3.0177E+02 | 4.1725E+00 |
| 25 | 2.1827E+02 | 3.0065E+02 | 2.2148E+02 | 2.2479E+02 | 1.5057E+01 |  | 2.9427E+02 | 3.2062E+02 | 3.1332E+02 | 3.1227E+02 | 4.9993E+00 |
| 26 | 2.0021E+02 | 3.5300E+02 | 3.2829E+02 | 3.2134E+02 | 3.4688E+01 |  | 2.0309E+02 | 4.0197E+02 | 3.8863E+02 | 3.1507E+02 | 9.1226E+01 |
| 27 | 5.4359E+02 | 1.1322E+03 | 8.6964E+02 | 8.6964E+02 | 1.3793E+02 |  | 1.0926E+03 | 1.3328E+03 | 1.2479E+03 | 1.2508E+03 | 4.6449E+01 |
| 28 | 3.0404E+03 | 5.4618E+03 | 4.3804E+03 | 4.3121E+03 | 5.4480E+02 |  | 2.4199E+03 | 3.2763E+03 | 2.5865E+03 | 2.6137E+03 | 1.4522E+02 |
|  |  |  |  |  |  |  |  |  |  |  |  |

**Supplementary text S1: Working of the Algorithm**

Evolutionary algorithms exhibit some common properties pertaining to their working principle. To show these properties or stages on our algorithm, we have taken a two dimensional Rastrigin function with global minima at {0, 0}. Supplementary Fig. S1 shows the three stages of evolutionary search, exhibiting, how the individuals are distributed in the beginning (S1.a), somewhere in the middle(S1.b) and at the end of the evolution(S1.c). As it can be observed, initially the individuals are distributed randomly over the search space. Later, they start converging towards local or global minima. This phase is often termed as “exploration”, in which the individuals start exploring the untested regions of search space. In the later stages of the process, the whole population concentrates around local or global minimum which is often termed as “exploitation”. In the figure we can clearly see that the whole population is now concentrated around {0, 0}. The individuals sort of restrict their search space around this region in order to fine tune their already found minimum.


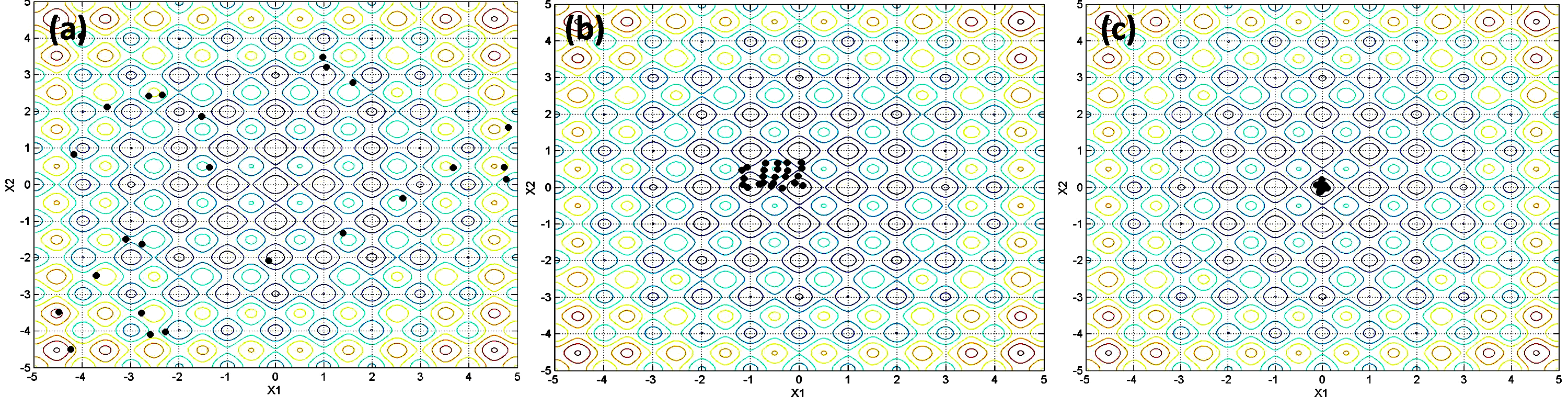


Figure S1: Illustration of different stages of evolutionary process of the algorithm. This figure shows the population distribution during a) Individualization stage b) Exploration stage c) Exploitation stage at 25, 100 and 10000 function evaluations, respectively. This experiment was performed on 2-D Rastrigin function with global minima at {0, 0}. Individuals are shown by black circles superimposed on the Rastrigin contour.

1. Liang, J. J., Qu, B. Y. & Suganthan, P. N. Problem Definitions and Evaluation Criteria for the CEC 2013 Special Session on Real-Parameter Optimization. (2013).
